# Long non-coding RNA SNHG22 facilitates the malignant phenotypes in triple-negative breast cancer via sponging miR-324-3p and upregulating SUDS3

**DOI:** 10.1186/s12935-020-01321-9

**Published:** 2020-06-17

**Authors:** Xuan Fang, Jin Zhang, Chunyan Li, Jinjin Liu, Zhendong Shi, Peng Zhou

**Affiliations:** 1 The Third Department of Breast Cancer, Tianjin Medical University Cancer Institute and Hospital, National Clinical Research Center for Cancer, West Huan-Hu Rd., Ti Yuan Bei, Hexi District, Tianjin, 300060 People’s Republic of China; 2 Key Laboratory of Breast Cancer Prevention and Therapy, Tianjin Medical University, Ministry of Education, Tianjin, People’s Republic of China; 3 Key Laboratory of Cancer Prevention and Therapy, Tianjin, People’s Republic of China; 4 Tianjin’s Clinical Research Center for Cancer, Tianjin, People’s Republic of China

**Keywords:** SNHG22, miR-324-3p, SUDS3, Triple-negative breast cancer

## Abstract

**Background:**

Increasing evidence has indicated the important role of long non-coding RNAs (lncRNAs) in regulating the development and progression of cancers, including triple-negative breast cancer (TNBC). Small nucleolar RNA host gene 22 (SNHG22) is a novel lncRNA that has been identified as tumor-contributor in ovarian carcinoma. However, its function has not been explored in TNBC.

**Methods:**

qRT-PCR was used to identify gene expression at mRNA level while western blot was utilized to analyze the protein level. Functional assays were implemented to identify changes on the proliferation, apoptosis and motility of TNBC cells under different conditions. Additionally, mechanistic assays, such as RIP assay, RNA pull down assay and luciferase reporter assay, were applied to assess relationships between molecules.

**Results:**

SNHG22 represented a high expression level in TNBC tissues and cells. Besides, SNHG22 silencing restrained the proliferation, migration and invasion of TNBC cells. Furthermore, miR-324-3p that was lowly expressed in TNBC cells was conformed to be sponged by SNHG22. Moreover, upregulated miR-324-3p inhibited cell proliferation and motility in TNBC. Subsequently, we identified that SUDS3, a tumor-facilitator with elevated expression in TNBC, was the downstream target of SNHG22/miR-324-3p axis. Of note, miR-324-3p repression or SUDS3 overexpression could rescue the anti-tumor effect of SNHG22 silencing on the malignant phenotypes of TNBC cells.

**Conclusion:**

LncRNA SNHG22 facilitated cell growth and motility in TNBC via sponging miR-324-3p and upregulating SUDS3, highlighting a new promising road for TNBC treatment development.

## Background

Breast cancer is a prevalent female cancer with a high incidence. As one of the most aggressive subtypes of breast cancer [1], triple-negative breast cancer (TNBC) accounts for about fifteen percent of all cases [2]. Patients with TNBC are featured by negative expression of both hormone receptor (HR) and human epidermal growth factor receptor-2 (HER-2) [3, 4]. With the development of treatment technology, the current main treatment methods for TNBC patients are surgery, chemotherapy and so on. Nevertheless, because of the aggressive nature, the prognosis of patients with TNBC is not good [5]. Therefore, exploring the pathogenesis and molecular mechanisms of TNBC can help find more effective therapeutic strategies.

In recent years, a flow of studies has been confirmed that long non-coding RNAs (lncRNAs) are RNA transcripts which have over 200 nucleotides in length and lack of ability to encode proteins [6]. LncRNAs can take part in a variety of biological processes such as cell proliferation, migration and apoptosis. What’s more, they have also been proved to be the crucial regulators during cancer development [7]. Increasing evidence has indicated that lncRNAs could affect the progression of cancers via regulating downstream genes through different mechanisms, including competing endogenous RNA (ceRNA) network [8, 9]. For example, FTX could sponge miR-215 and hinder Vimentin phosphorylation to accelerate colorectal cancer progression [10]. SPRY4-IT1 acted as a ceRNA to expedite cell growth in bladder cancer through upregulating EZH2 [11]. Moreover, PVT1 was reported to facilitate malignant phenotypes of pancreatic cancer cells via absorbing miR-448 [12]. Small nucleolar RNA host gene 22 (SNHG22) is a novel lncRNA which was discovered as highly expressed and as a tumor-promoter in epithelial ovarian carcinoma cells through the miR-2467/Gal-1 axis [13]. Nevertheless, the regulatory mechanism and function of SNHG22 in TNBC have not been elaborated.

In our research, the fundamental purpose was to inspect the function and probable mechanism of SNHG22 in TNBC, which might provide the new idea for TNBC treatment.

## Methods

### Tissue collection

Total 70 pairs of TNBC samples were acquired from Tianjin Medical University Cancer Institute and Hospital, and the study was implemented under the approval of Ethics Committee of Tianjin Medical University Cancer Institute and Hospital. Paired non-tumor tissues were defined as tissues at least five cm away from the tumor margin. After surgical resection, tissues were sharply frozen via liquid nitrogen and then maintained at − 80 °C until following use. Patients treated with radiotherapy or chemotherapy before surgery were excluded, and all of them had signed the written informed consent prior to this study.

### Cell lines

Human TNBC cell lines (MDA-MB-231, HCC-1937, MDA-MB-468 and MDA-MB-436) and normal breast epithelial cell line (MCF10A) used in this study were all procured from ATCC (Manassas, VA, USA) and then grown in DMEM (Gibco, Rockville, MD). The 10% FBS and 1% antibiotics were acquired from Gibco to supplement culture medium. Cells were all maintained under the condition of 5% CO_2_ and 37 °C.

### Total RNA isolation and qRT-PCR

TRIzol reagent was used to isolate the total RNA samples from cancer cells or tissues as per instruction (Invitrogen, Carlsbad, CA), and then cDNA synthesis was achieved with Reverse Transcription kit (Takara, Kyoto, Japan). SYBR GREEN PCR Master and ABI Prism 7900HT were purchased from Applied Biosystems (Foster City, CA) for quantitative analysis. Gene expression was appropriately relative to GAPDH or U6 based on 2^−ΔΔCt^ method. The primer sequences were shown as follows: SNHG22: CTAAGAGTGGCCTCTGCGTG (forward), CAAGGCACCTAACAGGGGAG (reverse); miR-324-3p: ATTAGCCCACTGCCCCAGGT (forward), CCCACTGCCCCAGGTGCTGCTGG (reverse); SUDS3: CTGCCGGTCCTCGAGTTG (forward), CACTCATGTCGCCTCTGACC (reverse); GAPDH: GGAGCGAGATCCCTCCAAAAT (forward), GGCTGTTGTCATACTTCTCATGG (reverse); U6: CTCGCTTCGGCAGCACA (forward), AACGCTTCACGAATTTGCGT (reverse).

### Cell transfection

The specific shRNAs against SNHG22 and corresponding negative control shRNAs, as well as the pcDNA3.1 vector containing SUDS3 and the empty vector (control), were all available from Genechem (Shanghai, China). Besides, the miR-324-3p mimics/inhibitor and NC mimics/inhibitor were synthesized by Ribobio (Guangzhou, China). MDA-MB-231 and MDA-MB-468 cells prepared in 24-well plates were subjected to transfection with appropriate plasmids above in the presence of Lipofectamine 3000 (Invitrogen). After 48 h of transfections, cells were collected for further use in following experiments.

### Colony formation assay

The MDA-MB-231 and MDA-MB-468 cells after 48 h of transfection were added to 6-well plates for 14 days of incubation. Resultantly, the formed clones were fixated by 4% formaldehyde and stained by 0.5% crystal violet. 5 min later, colonies containing more than 50 cells were imaged and counted manually.

### EdU assay

EdU assay were implemented in TNBC cells with BeyoClick™ EdU Cell Proliferation Kit with Alexa Fluor 594 as instructed (Beyotime, Shanghai, China). In brief, cells were rinsed in PBS and then processed with EdU solution for 2 h. Next, cells were fixed and re-stained by DAPI for nuclear detection, and then observed under inverted microscope (Olympus, Tokyo, Japan).

### Caspase-3 activity detection

Caspase-3 activity was estimated by Caspase-3 activity assay kit (Beyotime) according to the user manual. The cell protein extracts were cultivated with the caspase-3 substrate and assay buffer. Four hours later, the absorbance at 405 nm was analyzed via a microplate reader (Dynex Technologies, West Sussex, UK).

### Flow cytometry analysis

Cell apoptosis of TNBC cells was assayed by Annexin V/PI staining method (Invitrogen) as per specification. Cell samples were double-stained for 15 min in darkroom, and then subjected to analysis using the flow cytometry (BD Biosciences, Franklin Lakes, NJ).

### Wound healing assay

Cells were seeded to 6-well plates and grown to 90% confluence, and then the scratch was created in each well by a 200-μL pipette tip. The wound closure was imaged under Olympus microscope at 0 h when the scratch was created and at 24 h after being cultured in serum-free medium for 1 day. Finally, relative distance of wound healing was determined as the ratio of (the wound distance at 0 h—the wound distance at 24 h) to (the wound distance at 0 h).

### Cell invasion assay

The transwell chamber (8-mm pore size; Corning Incorporated, Big Flats, NY, USA) was pre-coated with 30 μg of Matrigel (BD Biosciences) for cell invasion assay. In short, cells (1 × 10^5^) in serum-free medium were added to the upper chamber. In the meantime, lower chamber was added with complete DMEM culture medium with 10% FBS. Following 24 h of culture at 37 °C with 5% CO_2_, cells invaded to the lower chamber were fixated and stained in crystal violet solution. At length, the invaded cells in 5 random fields were counted under an optical microscope (Thermo Fisher).

### Subcellular fractionation

The cytoplasmic and nuclear RNAs were isolated from cells by use of PARIS™ Kit (Invitrogen) in line with instruction. Cells were lysed in cell fractionation buffer, and then centrifuged and lysed in cell disruption buffer. SNHG22 expression was detected in both fractions using qRT-PCR, with GAPDH and U6 as cytoplasmic and nuclear controls, respectively.

### RNA immunoprecipitation (RIP)

RIP assay was carried out with the application of Magna RIP RNA-Binding Protein Immunoprecipitation Kit (EMD Millipore, Billerica, MA). TNBC cells were collected for lysing in RIP lysis buffer, and then the lysates were processed with human Ago2 antibody (Millipore) conjugated on magnetic beads, with IgG antibody as the control group. RNAs in immunoprecipitates were analyzed by qRT-PCR.

### RNA pull down assay

RNA pull down assay was studied by Pierce Magnetic RNA–Protein Pull-Down Kit (Thermo Fisher Scientific, Waltham, MA, USA). Firstly, Bio-miR-324-3p-WT/Mut containing wild-type or mutated seed regions, as well as their control Bio-NC, were obtained by using the Biotin RNA Labeling Mix (Roche, Mannheim, Germany) and T7 RNA polymerase (Roche). Subsequently, cell lysates collected using RIPA lysis buffer were subjected to above biotin-labeled miRNAs overnight. The magnetic beads coupled with streptavidin were afterwards added to pull down biotin-labeled miRNAs as well as their interacting compounds. Finally, RNAs (SNHG22 or SUDS3 mRNA) in the compounds of each group was assayed using qRT-PCR.

### Luciferase reporter assay

The SNHG22 or SUDS3 fragments covering the wild-type and mutated miR-324-3p binding sites were obtained for constructing SNHG22-WT/Mut and SUDS3-WT/Mut reporters employing pmirGLO dual-luciferase vector (Promega, Madison, WI, USA). Then, above constructs were individually co-transfected with miR-324-3p mimics or NC mimics into indicated TNBC cells for 48 h. At last, the relative luciferase activity was studied by luciferase reporter assay system (Promega).

### In vivo tumor growth model

4-week-old male BALB/c nude mice purchased from Shi Laike Company (Shanghai, China) were maintained in specific pathogen free (SPF)-grade animal lab. In vivo tumor growth was simulated via subcutaneously injecting mice with 1 × 10^6^ MDA-MB-231 cells transfected with sh-NC, sh-SNHG22#1, sh-SNHG22#1+miR-324-3p inhibitor or sh-SNHG22#1+SUDS3. Tumor volume was recorded every 4 days. Four weeks later, mice were sacrificed and tumors were weighted. Animal studies were approved by the Animal Ethics Committee of Tianjin Medical University Cancer Institute and Hospital.

### Immunohistochemistry (IHC)

The tumor tissues acquired from in vivo assays were fixed in 4% paraformaldehyde, dehydrated, and embedded in paraffin, in succession. Then, paraffin-embedded tissues were cut into 4-mm-thick sections. After being de-paraffinized, the sections were processed with antibodies against Ki67 (Abcam) overnight at 4 °C and subsequently with secondary antibody for 30 min at 37 °C. Thereafter, sections were subjected to hematoxylin for nuclear staining, and finally observed and photographed under a microscope.

### Statistical analyses

Each assay was repeated separately for three times, and experimental data were given with mean ± S.D. after processed by Prism 6.0 software (GraphPad Software, Inc., La Jolla, CA). The differences between groups were analyzed via Student’s t-test or one-way ANOVA, and data were thought significantly different when P-value below 0.05.

## Result

### SNHG22 was strongly expressed in TNBC cells and facilitated TNBC cell growth and motility

**Fig. 1:**
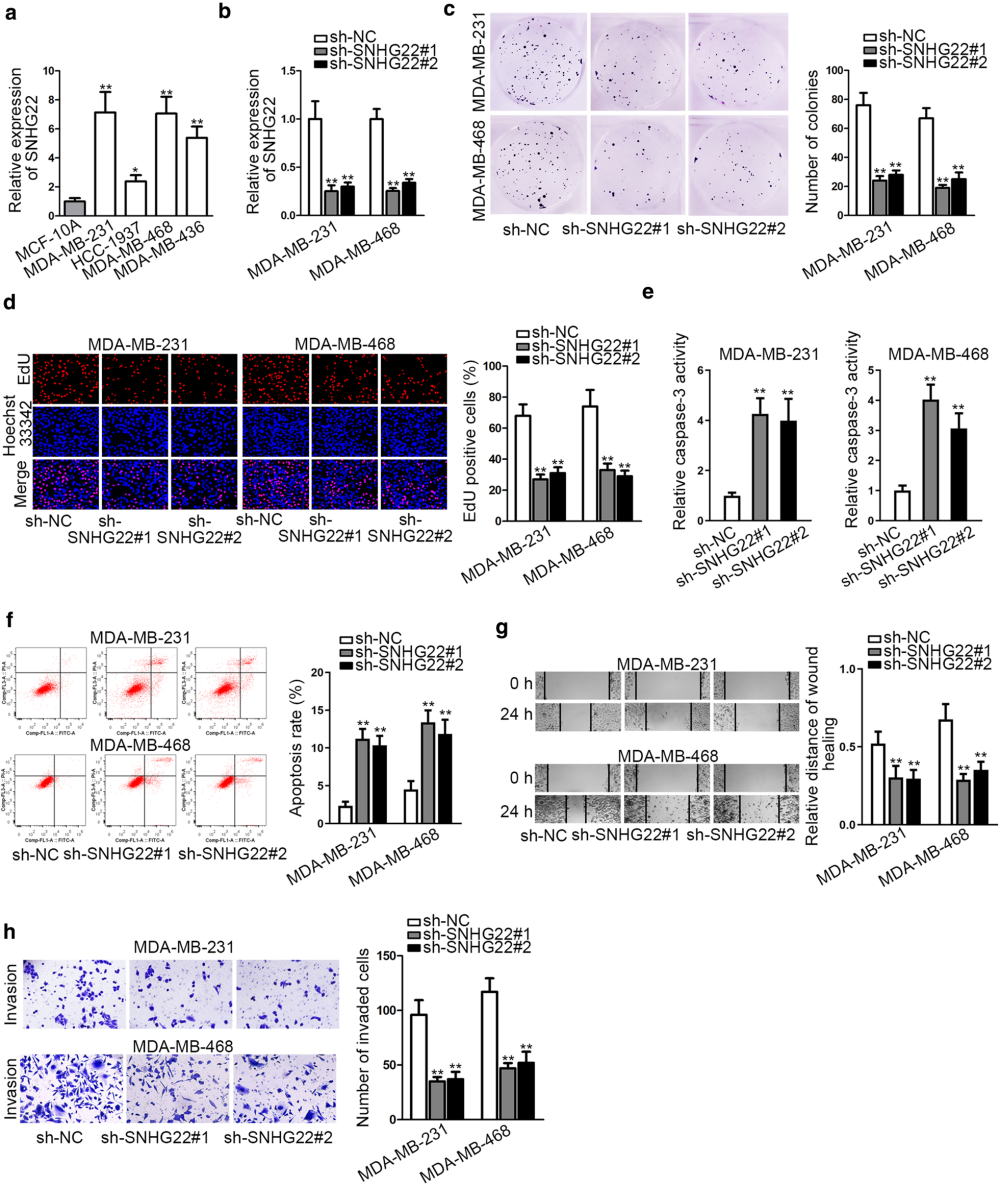
SNHG22 was highly expressed in TNBC cells and facilitated TNBC cell growth. **a** The expression of SNHG22 was tested in TNBC cells and the normal MCF-10A cells through qRT-PCR. **b** The knockdown efficiency of SNHG22 was detected through qRT-PCR in MDA-MB-231 and MDA-MB-468 cells. **c**, **d** Colony formation and EdU experiments were utilized to measure cell proliferation after knockdown of SNHG22. **e**, **f** Caspase-3 detection and flow cytometry analysis were utilized to estimate cell apoptosis in these two TNBC cells after silencing SNHG22. **g**, **h** Wound healing and transwell experiments were conducted to evaluate cell migration and invasion when SNHG22 was inhibited in indicated TNBC cells. *P < 0.05, **P < 0.01

For the sake of searching the functions of SNHG22 in TNBC, we firstly wondered whether its expression changed in malignant conditions. We discovered that SNHG22 expression was extremely strong in TNBC cell lines relative to MCF-10A cells, particularly in MDA-MB-231 and MDA-MB-468 cells (Fig. 1a). Therefore, we implemented loss-of-function experiments in MDA-MB-231 and MDA-MB-468 cells who had higher original SNHG22 expression to investigate the influence of SNHG22 on TNBC cell function. As observed in Fig. 1b, SNHG22 expression in these two cells was visibly weakened after transfection of two shRNAs aiming at SNHG22. Expectedly, it was displayed that the colony formation ability and EdU-positive cell proportion were both declined after silencing SNHG22 (Fig. 1c, d), indicating that knockdown of SNHG22 evidently impaired TNBC cell proliferation. By contrast, we discovered that the relative caspase-3 activity was intensified in two TNBC cells when SNHG22 was inhibited (Fig. 1e), indicating that SNHG22 deficiency could expedite cell apoptosis. Then the apoptosis-accelerating impact of SNHG22 suppression in TNBC cells was further evidenced by flow cytometry analysis (Fig. 1f). Moreover, we unveiled the restrained migration and invasion of the two TNBC cells in response to SNHG22 deficiency (Fig. 1g, h). Overall, SNHG22 was highly expressed in TNBC cells and facilitated TNBC cell proliferation and motility.

### SNHG22 acted as miR-324-3p sponge in TNBC

**Fig. 2:**
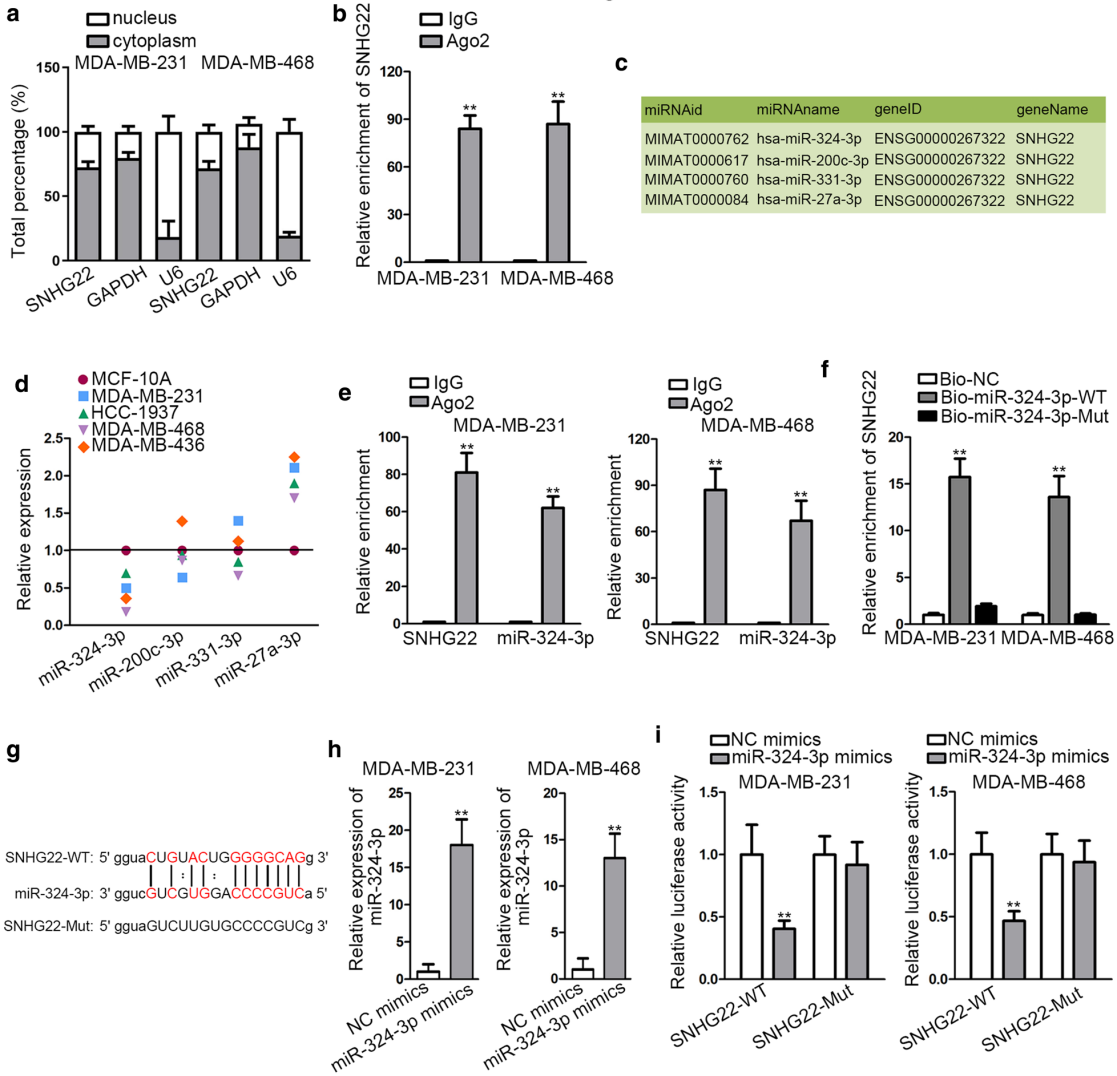
SNHG22 acted as miR-324-3p sponge in TNBC. **a** The distribution of SNHG22 in MDA-MB-231 and MDA-MB-468 cells was detected through subcellular fractionation assay. **b** RIP assay was implemented to verify whether SNHG22 could exist in RISCs. **c** The possible miRNAs interacted with SNHG22 were predicted from ENCORI under the condition of pan-cancer ≥ 6. **d** The expression levels of miR-324-3p, miR-200c-3p, miR-331-3p and miR-27a-3p in TNBC cells were tested through qRT-PCR. **e**, **f** RIP and RNA pull down experiments were carried out to evaluate the binding situation of miR-324-3p and SNHG22. **g** The binding sites between miR-324-3p and SNHG22 obtained from ENCORI. **h** The overexpression efficiency of miR-324-3p was tested by qRT-PCR. **i** Luciferase reporter experiment was utilized to prove the interaction of miR-324-3p with SNHG22. **P < 0.01

It has been confirmed through many reports that lncRNAs can function as a ceRNA to release mRNAs by sponging miRNAs in the cytoplasm of cells [14]. Thus, we investigated whether SNHG22 could directly bind with miRNA by acting as a ceRNA in TNBC. Luckily, it was indicated that SNHG22 was prominently located in the cytoplasm of MDA-MB-231 and MDA-MB-468 cells (Fig. 2a). Further, RIP assay demonstrated that SNHG22 was largely concentrated in Ago2 group rather than in IgG group, indicating the existence of SNHG22 in Ago2-comprised RNA-induced silencing complexes (RISCs) (Fig. 2b). On this basis, we guessed that SNHG22 might function in TNBC via sponging certain miRNAs. Thus, we utilized ENCORI (http://starbase.sysu.edu.cn/index.php) to find out the possible miRNAs (pan-cancer ≥ 6) which could bind to SNHG22. Fortunately, four miRNAs (miR-324-3p, miR-200c-3p, miR-331-3p and miR-27a-3p) were discovered (Fig. 2c). To further screen out the suitable miRNA involved in SNHG22-affected TNBC development, we implemented qRT-PCR to test the expression of above four candidates in TNBC cells and the normal MCF-10A cells. The results displayed the most down-regulation expression of miR-324-3p in TNBC cell lines while others showed no expression difference or high expression pattern (Fig. 2d). Thus, we selected miR-324-3p as the possible miRNA downstream of SNHG22 in TNBC. Intriguingly, the results of RIP assays depicted that both of miR-324-3p and SNHG22 were enriched massively in Ago2 group (Fig. 2e). Moreover, SNHG22 was harvested in the complexes pulled down by biotinylated miR-324-3p-WT rather than the mutated type (Fig. 2f), highlighting the binding of miR-324-3p to SNHG22 via its seed region. Thereafter, the binding sites between miR-324-3p and SNHG22 was predicted by ENCORI (Fig. 2g). After that, we overexpressed miR-324-3p in MDA-MB-231 and MDA-MB-468 cells (Fig. 2h). Expectedly, it was verified that miR-324-3p enhancement could lead to the notable reduction on the luciferase activity of SNHG22-WT, but not that of SNHG22-Mut (Fig. 2i). All of these experiments indicated that SNHG22 acted as miR-324-3p sponge in TNBC.

### MiR-324-3p restrained the malignant behaviors of TNBC cells

**Fig. 3:**
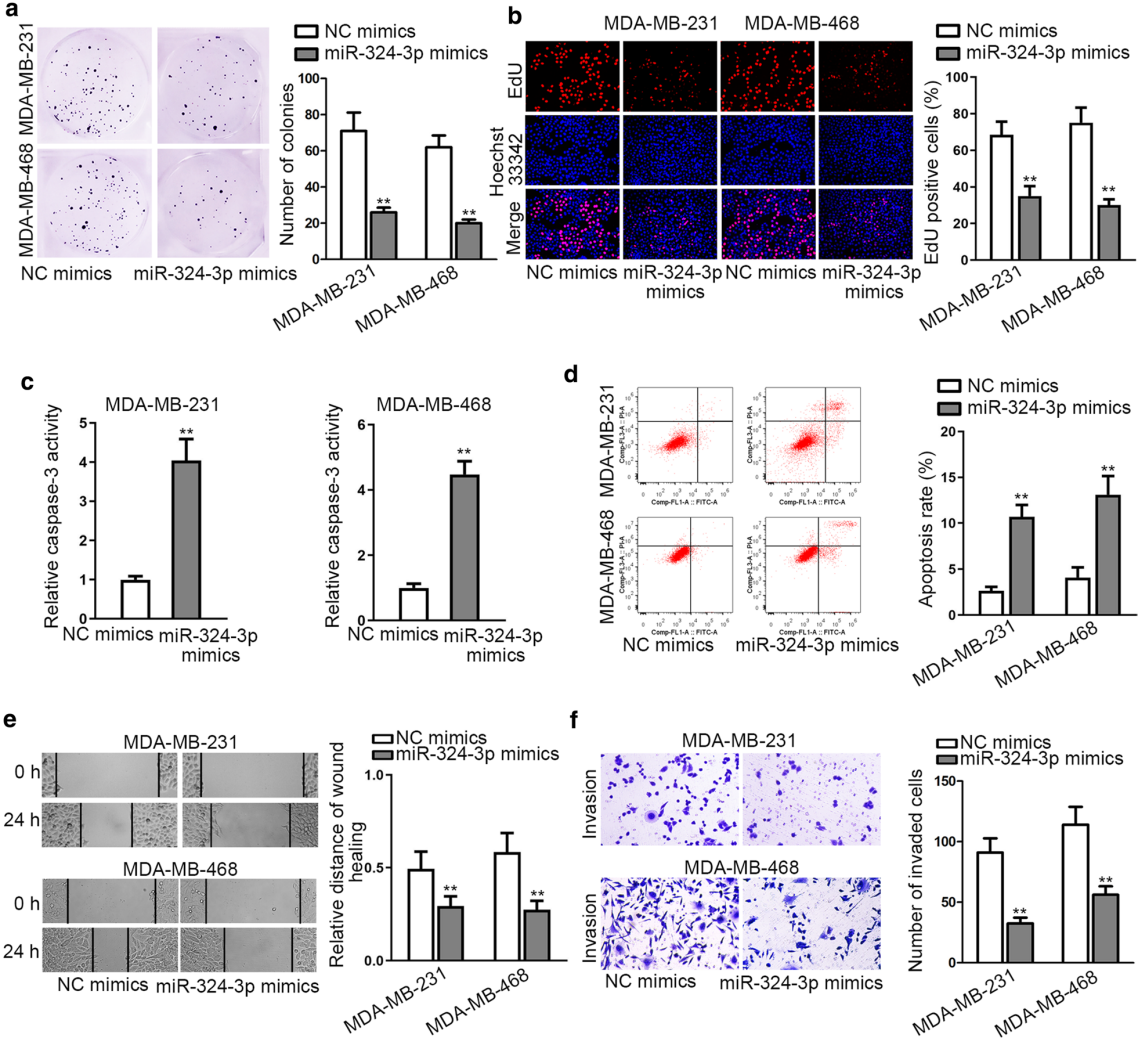
MiR-324-3p restrained the progression of TNBC. **a**, **b** The proliferation of MDA-MB-231 and MDA-MB-468 cells with or without elevated miR-324-3p expression was measured through colony formation and EdU experiments. **c**, **d** Caspase-3 detection and flow cytometry experiments were utilized to evaluate the apoptosis in above MDA-MB-231 and MDA-MB-468 cells. **e**, **f** Cell migration and invasion capabilities were estimated by wound healing assay and transwell experiments in above TNBC cells. **P < 0.01

Next, we planned to research the biological function of miR-324-3p in TNBC. Results indicated that overexpressed miR-324-3p reduced the number of colonies and the proportion of EdU positive cells, which meat blocked proliferative ability of TNBC cells in face of miR-324-3p overexpression (Fig. 3a, b). Then we observed that caspase-3 activity was evidently heightened and the apoptosis rate was accelerated after upregulating miR-324-3p, indicating that cell apoptosis was promoted by miR-324-3p overexpression (Fig. 3c, d). In the end, through wound healing and transwell assays, we discovered that cell motility was impaired owing to ectopic expression of miR-324-3p (Fig. 3e, f). Taken together, miR-324-3p restrained the malignant behaviors of TNBC cells.

### MiR-324-3p targeted SUDS3 in TNBC

**Fig. 4:**
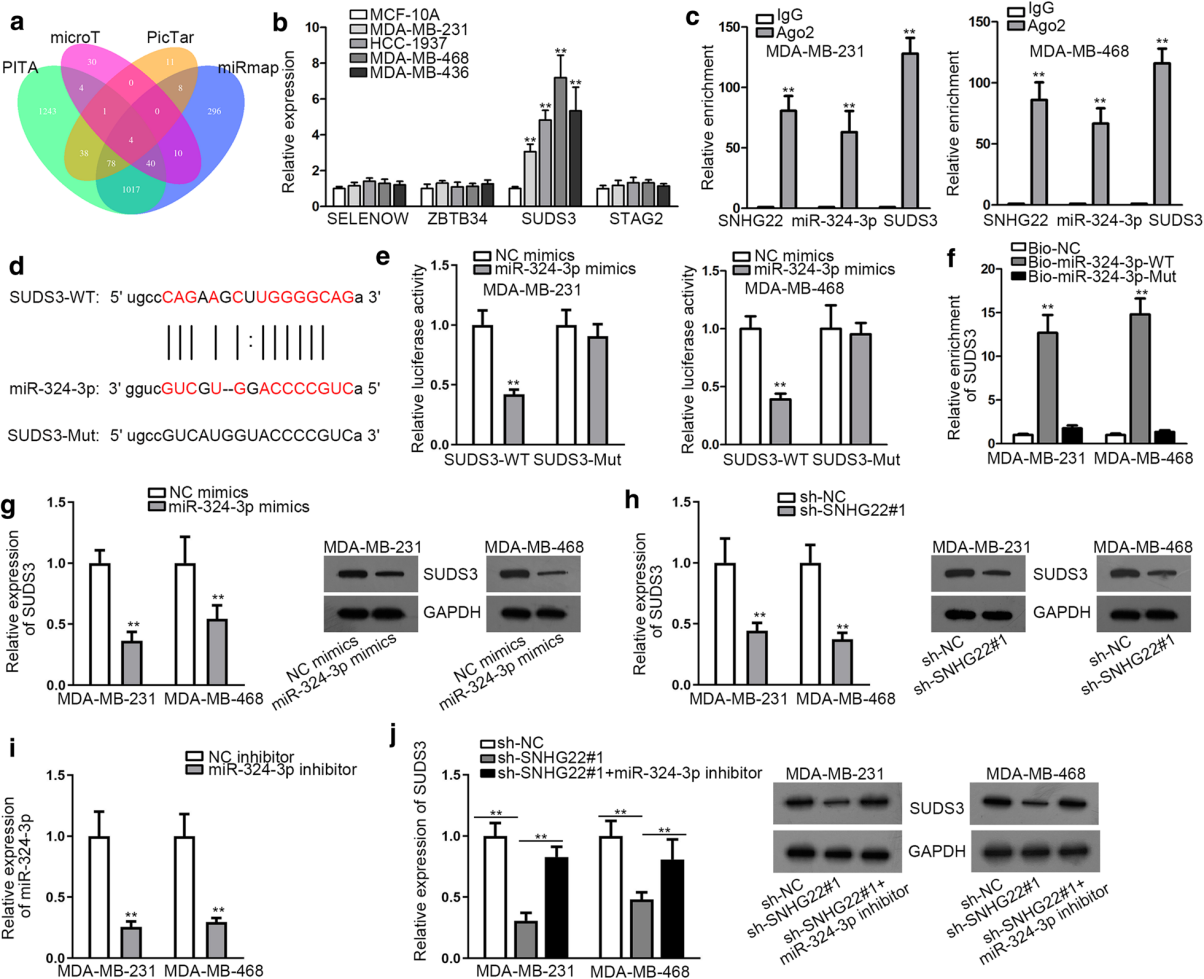
MiR-324-3p targeted SUDS3 in TNBC. **a** The possible mRNAs bound to miR-324-3p were predicted by PITA, microT, PicTar and miRmap databases. **b** The expression levels of SELENOW, ZBTB34, SUDS3 and STAG2 were tested through qRT-PCR in TNBC cells and MCF-10A cells. **c** RIP experiment was utilized to test the co-harvest of SNHG22, miR-324-3p and SUDS3 in RISCs. **d** The binding sites between miR-324-3p and SUDS3 provided by ENCORI. **e**, **f** Luciferase reporter and RNA pull down experiments were conducted to verify the correlation between miR-324-3p and SUDS3. **g**, **h** The expression of SUDS3 was tested by qRT-PCR and western blot when miR-324-3p was overexpressed or SNHG22 was inhibited in MDA-MB-231 and MDA-MB-468 cells. **i** The interference efficiency of miR-324-3p was detected through qRT-PCR in MDA-MB-231 and MDA-MB-468 cells. **j** The expression of SUDS3 was tested through qRT-PCR and western blot in MDA-MB-231 and MDA-MB-468 cells with inhibited SNHG22 or together with suppressed miR-324-3p. **P < 0.01

For the sake of further searching the regulatory mechanism, we continued to figure out the target of miR-324-3p. Under predictions of microT, PicTar, miRmap and PITA tools, four potential mRNAs (SELENOW, ZBTB34, SUDS3 and STAG2) that were predicted by all these four tools were selected, while those predicted by only one, two or three tools were excluded (Fig. 4a). Then qRT-PCR analysis revealed that SUDS3 expression was greatly enhanced in TNBC cells in comparison with MCF-10A cells, whereas the level of other genes hardly changed (Fig. 4b). More importantly, we discovered that when SUDS3 was silenced in MDA-MB-231 and MDA-MB-468 cells (Additional file 1: Figure S1A), the proliferation of them was hampered (Additional file 1: Figure S1B, C), the apoptosis was facilitated (Additional file 1: Figure S1D, E) and the motility was blocked (Additional file 1: Figure S1F, G). Based on these results, we selected SUDS3, a tumor-promoter in TNBC, as the downstream of SNHG22/miR-324-3p axis. To analyze the association of SUDS3 with SNHG22 and miR-324-3p in TNBC, we then implemented a series of experiments. RIP assay manifested that SNHG22, miR-324-3p and SUDS3 were all easily captured in Ago2 group, which indicated that they were co-existed in RISCs (Fig. 4c). Then the possible binding sites between miR-324-3p and SUDS3 were predicted from ENCORI and displayed in Fig. 4d. Later, luciferase reporter experiment validated that miR-324-3p overexpression considerably impaired the luciferase activity of SUDS3-WT rather than that of SUDS3-Mut (Fig. 4e). Furthermore, it was indicated that SUDS3 was specifically captured by Bio-miR-324-3p-WT (Fig. 4f). Furthermore, we discovered that SUDS3 expression at mRNA and protein levels could be inhibited by miR-324-3p overexpression and SNHG22 depletion (Fig. 4g, h), demonstrating that SUDS3 was negatively associated with miR-324-3p and positively associated with SNHG22. Then we confirmed he interference efficiency of miR-324-3p in these two cells through qRT-PCR (Fig. 4i). As expected, we unveiled that SUDS3 expression reduced by SNHG22 knockdown were then recovered by miR-324-3p inhibition, at both mRNA and protein levels (Fig. 4j). All of these experiments indicated that SUDS3 was targeted by miR-324-3p.

### SNHG22/miR-324-3p/SUDS3 axis contributed to TNBC cell growth

**Fig. 5:**
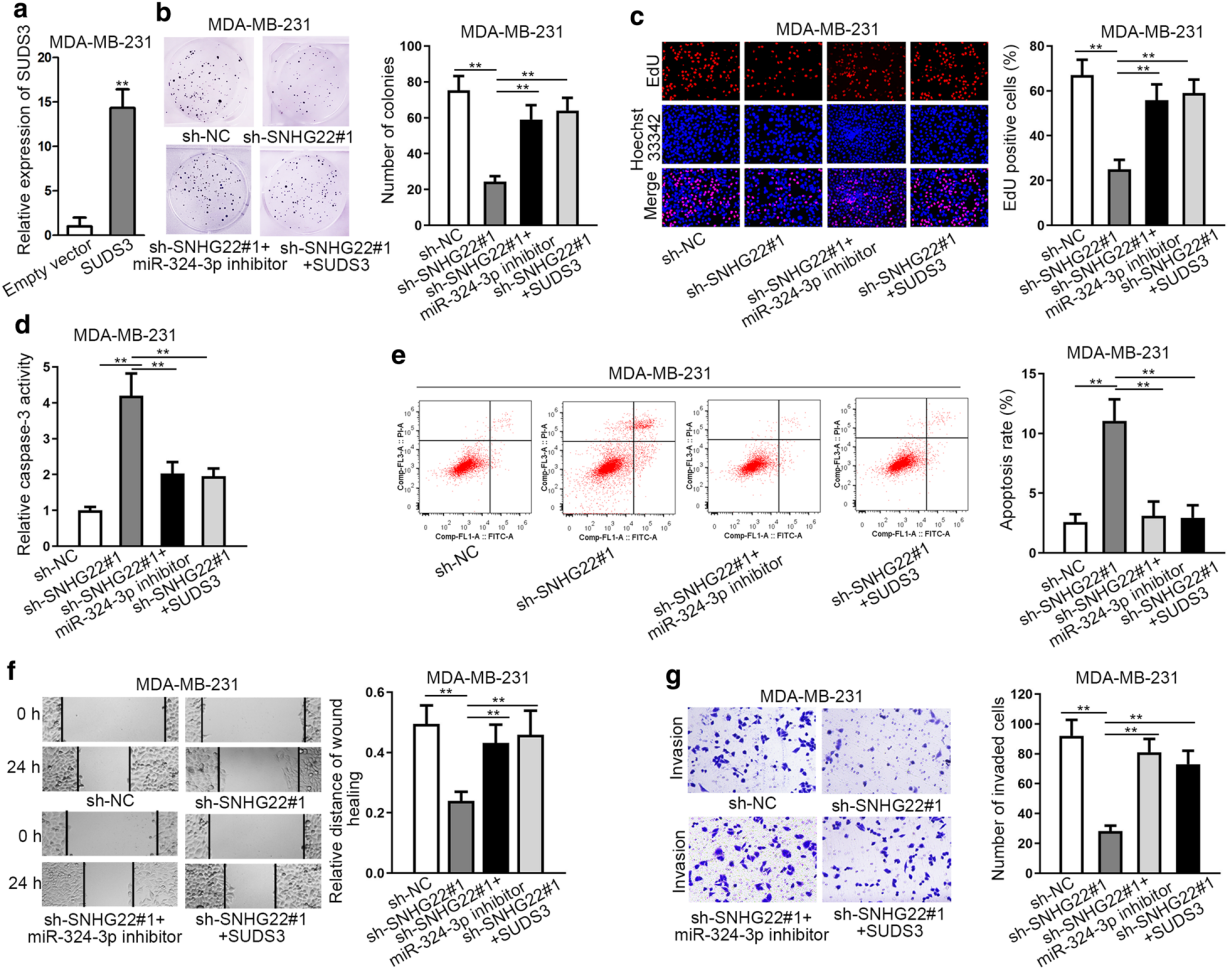
SNHG22/miR-324-3p/SUDS3 axis contributed to malignant behaviors of TNBC cells. **a** The overexpression efficiency of SUDS3 was tested in MDA-MB-231 cells through qRT-PCR. **b**, **c** Cell proliferation was evaluated by colony formation and EdU experiments in different groups. **d**, **e** Caspase-3 detection and flow cytometry experiments were implemented to estimate cell apoptosis in different groups. **f**, **g** Wound healing and transwell assays were carried out to test cell migration and invasion capabilities in different groups. **P < 0.01

For the sake of probing into the function of SNHG22/miR-324-3p/SUDS3 axis in TNBC, we carried out a series of restoration experiments. Prior to that, we validated the overexpression efficiency of SUDS3 through qRT-PCR in MDA-MB-231 cells (Fig. 5a). Then, SNHG22 depletion-suppressed cell proliferation was discovered to be reversed by either miR-324-3p inhibition or SUDS3 overexpression (Fig. 5b, c). On the contrary, we also observed that cell apoptosis accelerated by silencing SNHG22 was then abrogated by inhibited miR-324-3p or upregulated SUDS3 (Fig. 5d, e). Meanwhile, the suppression of silencing SNHG22 on cell motility was counteracted in the contexts of miR-324-3p restraint or SUDS3 upregulation (Fig. 5f, g). To sum up, SNHG22/miR-324-3p/SUDS3 axis contributed to the malignancy of TNBC both in vitro and in vivo.

### The role of SNHG22/miR-324-3p/SUDS3 axis in TNBC cell growth in vivo

**Fig. 6:**
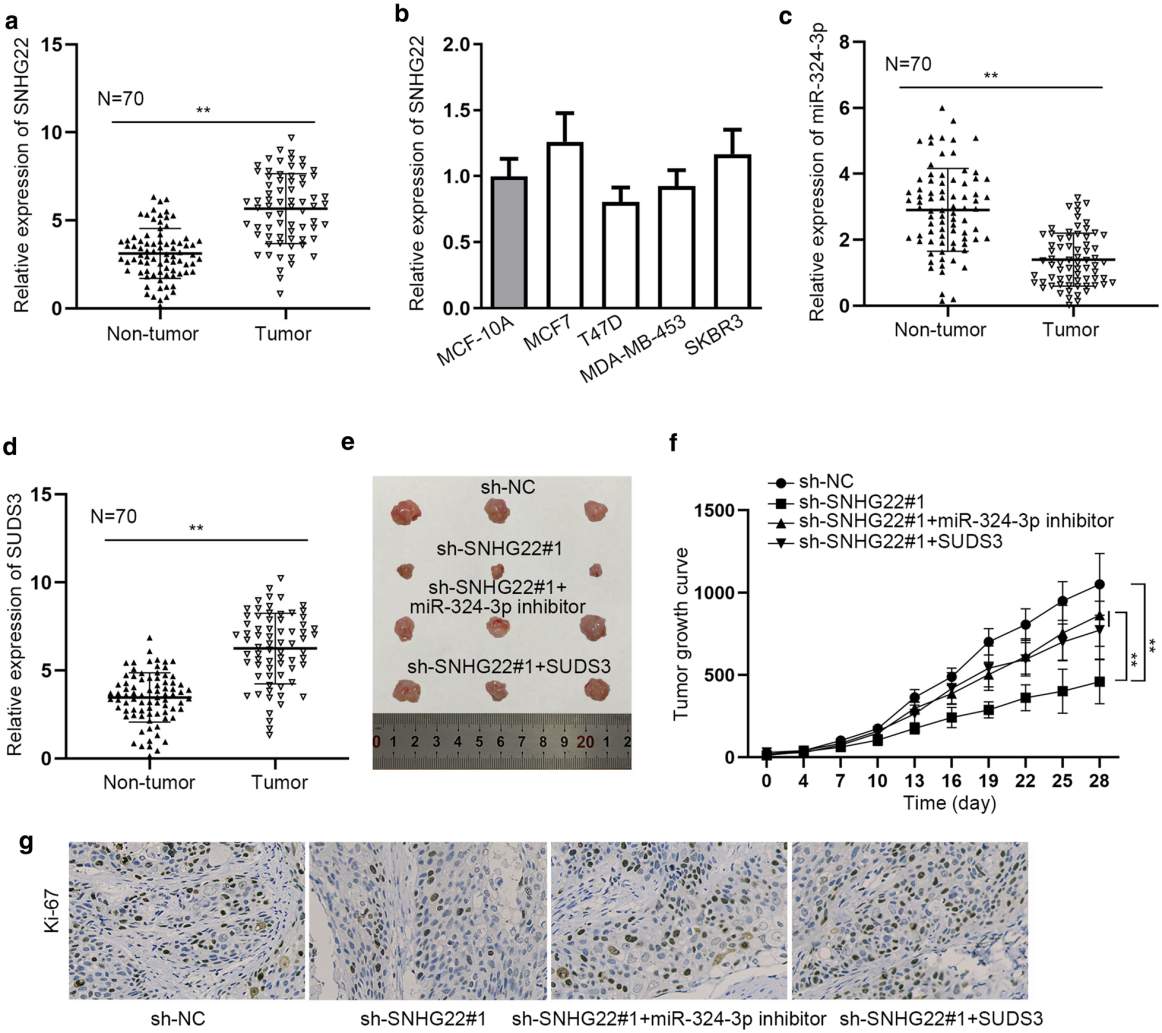
The role of SNHG22/miR-324-3p/SUDS3 axis in TNBC cell growth in vivo. **a** The expression of SNHG22 in TNBC tissues and paired non-tumor tissues was analyzed via qRT-PCR. **b** SNHG22 expression in cell lines belonging to other breast cancer subtypes compared to normal MCF-10A cells was determined by qRT-PCR. **c**, **d** qRT-PCR detected the expression of miR-324-3p and SUDS3 in pairs of clinical tissues. **e** Representative images of tumors originated from different MDA-MB-231 cells. **f** The growth curve of above tumors. **g** IHC assay detected Ki67 staining in tumors from different groups. **P < 0.01

To determine the importance of SNHG22/miR-324-3p/SUDS3 axis in TNBC, we further detected the expression level of them in clinical TNBC samples and conducted animal study. As a result, we discovered the high expression trend of SNHG22 in clinical TBC tissues relative to paired non-cancerous ones (Fig. 6a). Interestingly, it manifested that the expression of SNHG22 had no significant differentials between MCF-10A cells and hormone dependent breast cancer cell lines (MCF7 and T47D) or HER2-positive breast cancer cell lines (MDA-MB-453 and SKBR3) (Fig. 6b). In this regard, we speculated that SNHG22 might play a specific part in TNBC. Besides, we unveiled the lowered miR-324-3p level in TNBC samples relative to matched non-tumor ones (Fig. 6c). Also, heightened level of SUDS3 was verified in TNBC tumors in comparison to matched para-tumors (Fig. 6d). More importantly, the in vivo data also proved that SNHG22 depletion hindered TNBC cell growth but this effect could be offset by reduced miR-324-3p or elevated SUDS3 expression (Fig. 6e, f). Such macroscopic phenomena were accompanied by the microcosmic observations that lessened Ki67 staining in tumors with SNHG22 inhibition was also normalized under miR-324-3p inhibition or SUDS3 overexpression (Fig. 6g).

## Discussion

In recent years, increasing researches have suggested that lncRNAs could regulate the occurrence and development of assorted cancers, including TNBC. For example, PVT1 was reported to regulate TNBC progression by KLF5/beta-catenin signaling [15]. And C-MYC-induced upregulation of SNHG12 could accelerate cell growth of TNBC [16]. Moreover, lncRNA HCP5 expedited TNBC cell proliferation by serving as a ceRNA to regulate BIRC3 [17]. In our work, SNHG22 was utilized to be our research object. In accordance with previous research, SNHG22 was proved to be highly expressed and could induce chemotherapy resistance through miR-2467/Gal-1 axis in epithelial ovarian carcinoma [13]. In our research, we detected abnormally strong expression of SNHG22 in TNBC tissues and cells. Intriguingly, SNHG22 had specific upregulation in TNBC cells whereas had no observable differential expression in other breast cancer types. This finding highly suggested the specific role of SNHG22 in TNBC. Further, we unveiled that SNHG22 could facilitate cell proliferation, migration and invasion in TNBC, demonstrating the carcinogenic property of SNHG22 in TNBC.

MiRNAs are single-stranded RNAs and contain 22–24 nucleotides in length [18]. Considerable number of evidences prove lncRNAs as a ceRNA to boost the downstream mRNAs via sponging miRNAs [19, 20]. The ceRNA network has been discovered to have close relation to cancer development. For instance, MT1JP functioned as a ceRNA to regulate FBXW7 in gastric cancer via sequestering miR-92a-3p [21]. Of note, whether a lncRNA can function as a ceRNA depends on its subcellular localization. Mounting literatures have evidenced that cytoplasmic lncRNAs can serve as a ceRNA to affect protein-coding genes at post-transcriptional level [22].

In our research, we verified that SNHG22 was mainly located in cytoplasm of TNBC cells. Moreover, we recognized miR-324-3p as the downstream molecule of SNHG22 in TNBC. Previous research indicated that miR-324-3p suppressed nasopharyngeal carcinoma cell migration and invasion through silencing WNT2B [23]. In our work, miR-324-3p was revealed to have low expression tendency and can restrain cell proliferation and motility in TNBC. Furthermore, we found SUDS3 was targeted by miR-324-3p and that SNHG22 could regulate SUDS3 by miR-324-3p-mediated manner. More importantly, SUDS3 was negatively regulated by miR-324-3p and positively modulated by SNHG22 in TNBC. In addition, we disclosed that SUDS3 was highly expressed in TNBC, and that SUDS3 suppression led to mitigated malignancy in TNBC. Rescue assays validated that the restraining effect of SNHG22 depletion on TNBC progression was offset by miR-324-3p inhibition or SUDS3 overexpression. Lack of diagnostic and prognostic analysis is the limitation of our current study. We will explore the prognostic value of SNHG22/miR-324-3p/SUDS3 in TNBC.

## Conclusion

To sum up, our work proved that SNHG22 had a strong expression in TNBC and exerted carcinogenic effects in TNBC. Additionally, SNHG22 could facilitate TNBC progression via sequestering miR-324-3p and then upregulating SUDS3. Significantly, our findings might offer a new idea for the exploration of TNBC treatment. Specifically, considering its regulation on TNBC via miR-324-3p/SUDS3 axis, SNHG22 could serve as a promising molecular target for the development of targeted therapy in TNBC.

## Supplementary information

**Additional file 1: Figure S1.** (A) qRT-PCR tested the knockdown efficiency of SUDS3 in MDA-MB-231 and MDA-MB-468 cells. (B-C) Colony formation and EdU assays examined the proliferation of these two cells with or without SUDS3 inhibition. (D-E) The apoptosis of indicated cells was assayed via caspase-3 activity and flow cytometry analyses. (F-G) Transwell assay estimated the impact of SUDS3 inhibition on cell migration and invasion in MDA-MB-231 and MDA-MB-468 cells. ^**^P < 0.01.

## Data Availability

Not applicable.

## Abbreviations

lncRNAs: Long noncoding RNAs
mRNAs: Messenger RNAs
miRNAs: MicroRNAs
ceRNA: Competing endogenous RNA
TNBC: Triple-negative breast cancer
SNHG22: Small nucleolar RNA host gene 22
ATCC: American Type Culture Collection
FBS: Fatal Bovine Serun
DMEM: Dulbecco’s Modified Eagle Medium
qRT-PCR: Quantitative real-time PCR
EdU: 5-ethynyl-2′-deoxyuridine
RIP: RNA immunoprecipitation
WT: Wild-type
Mut: Mutant
S.D.: Standard deviation
ANOVA: Analysis of Variance
