# Supplementary figures and images for: Long non-coding RNA SNHG22 facilitates the malignant phenotypes in triple-negative breast cancer via sponging miR-324-3p and upregulating SUDS3
Source: Cancer Cell Int. 2020 Jun 17;20:252. doi: 10.1186/s12935-020-01321-9 (PMC7302359; doi:10.1186/s12935-020-01321-9)

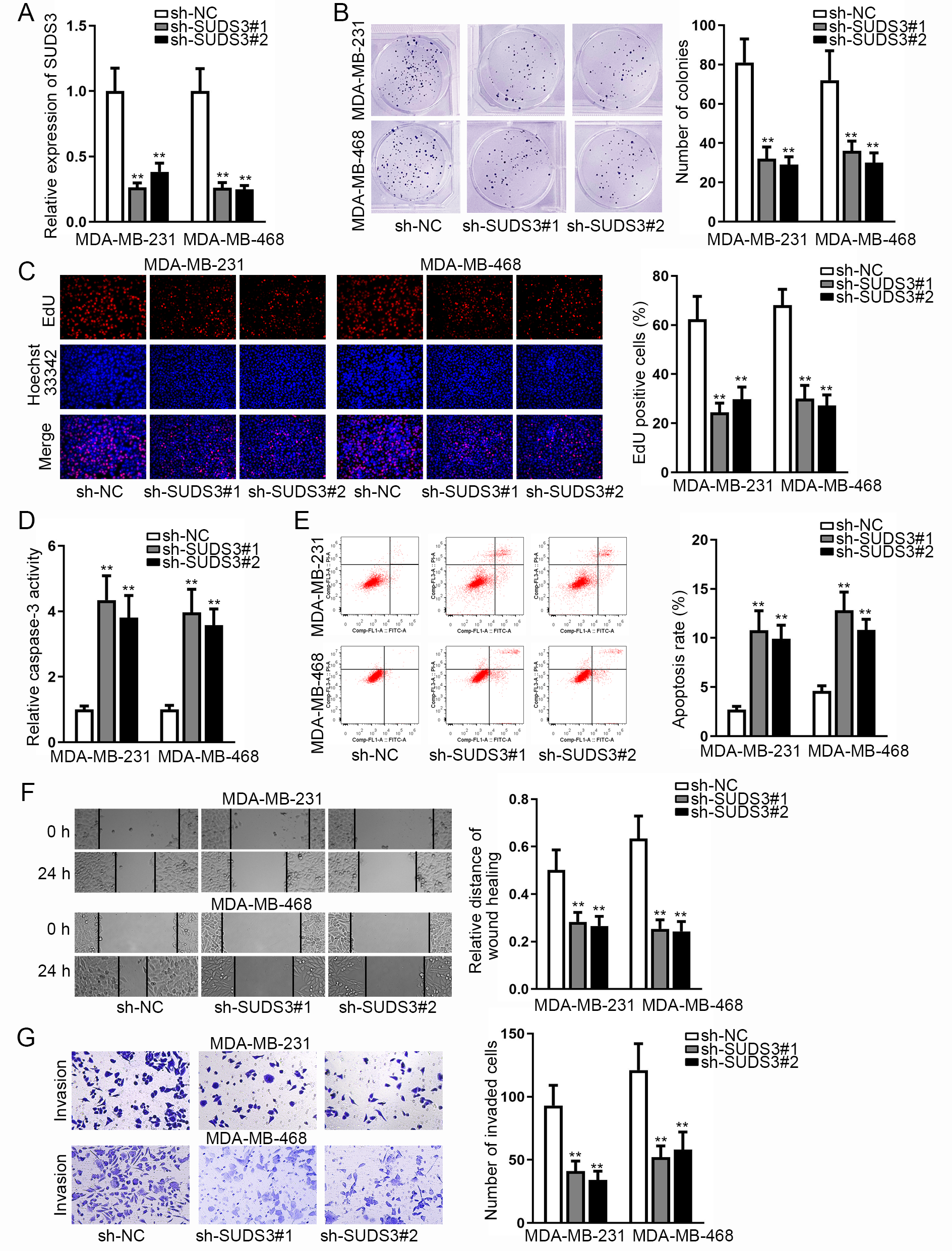

Supplement: Supplementary file 1 — Additional file 1: Figure S1. (A) qRT-PCR tested the knockdown efficiency of SUDS3 in MDA-MB-231 and MDA-MB-468 cells. (B-C) Colony formation and EdU assays examined the proliferation of these two cells with or without SUDS3 inhibition. (D-E) The apoptosis of indicated cells was assayed via caspase-3 activity and flow cytometry analyses. (F-G) Transwell assay estimated the impact of SUDS3 inhibition on cell migration and invasion in MDA-MB-231 and MDA-MB-468 cells. **P < 0.01. [file 12935_2020_1321_MOESM1_ESM.tif]
